# Scalable Bio Marker Combinations for Early Stroke Diagnosis: A Systematic Review

**DOI:** 10.3389/fneur.2021.638693

**Published:** 2021-05-28

**Authors:** Saiyet de la C. Baez, Diana García del Barco, Anette Hardy-Sosa, Gerardo Guillen Nieto, Maria Luisa Bringas-Vega, Jorge J. Llibre-Guerra, Pedro Valdes-Sosa

**Affiliations:** ^1^The Clinical Hospital of Chengdu Brain Sciences Institute, University Electronic Sciences and Technology of China UESTC, Chengdu, China; ^2^Center for Genetic Engineering and Biotechnology, Havana, Cuba; ^3^Cuban Neurosciences Center, Havana, Cuba; ^4^Department of Neurology, National Institute of Neurology and Neurosurgery of Cuba, Havana, Cuba; ^5^Department of Neurology, Washington University School of Medicine in St. Louis, St. Louis, MO, United States

**Keywords:** stroke, diagnosis, biomarker panels, serum biomarkers, neuroprotection

## Abstract

**Background:** Acute stroke treatment is a time-critical process in which every minute counts. Laboratory biomarkers are needed to aid clinical decisions in the diagnosis. Although imaging is critical for this process, these biomarkers may provide additional information to distinguish actual stroke from its mimics and monitor patient condition and the effect of potential neuroprotective strategies. For such biomarkers to be effectively scalable to public health in any economic setting, these must be cost-effective and non-invasive. We hypothesized that blood-based combinations (panels) of proteins might be the key to this approach and explored this possibility through a systematic review.

**Methods:** We followed the PRISMA (Preferred Reporting Items for Systematic Reviews and Meta-Analysis) guidelines for systematic review. Initially, the broader search for biomarkers for early stroke diagnosis yielded 704 hits, and five were added manually. We then narrowed the search to combinations (panels) of the protein markers obtained from the blood.

**Results:** Twelve articles dealing with blood-based panels of protein biomarkers for stroke were included in the systematic review. We observed that NR2 peptide (antibody against the NR2 fragment) and glial fibrillary acidic protein (GFAP) are brain-specific markers related to stroke. Von Willebrand factor (vWF), matrix metalloproteinase 9 (MMP-9), and S100β have been widely used as biomarkers, whereas others such as the ischemia-modified albumin (IMA) index, antithrombin III (AT-III), and fibrinogen have not been evaluated in combination. We herein propose the following new combination of biomarkers for future validation: panel 1 (NR2 + GFAP + MMP-9 + vWF + S100β), panel 2 (NR2 + GFAP + MMP-9 + vWF + IMA index), and panel 3 (NR2 + GFAP + AT-III + fibrinogen).

**Conclusions:** More research is needed to validate, identify, and introduce these panels of biomarkers into medical practice for stroke recurrence and diagnosis in a scalable manner. The evidence indicates that the most promising approach is to combine different blood-based proteins to provide diagnostic precision for health interventions. Through our systematic review, we suggest three novel biomarker panels based on the results in the literature and an interpretation based on stroke pathophysiology.

## Background

Stroke remains to be the second leading cause of death worldwide, with a yearly death toll of 5.5 million (1, 2). Furthermore, approximately 116.4 million people are reportedly disabled because of stroke, resulting in stroke being one of the most important causes of disability in older people (3). Consequently, cerebrovascular diseases have substantial economic impact and significant social consequences. This impact is exacerbated in lower- and middle-income countries. Evidence suggests that this situation is due to insufficient and non-optimal strategies for the prevention of cerebrovascular diseases and due to reduced availability of equipment for the diagnosis and treatment in medical centers (4).

Many of the shortcomings in managing stroke and related diseases are due to the heterogeneity of these pathologies. The main subtypes of stroke are ischemic and hemorrhagic stroke. Ischemic stroke is characterized by a lack of blood supply to a part of the brain, whereas hemorrhagic stroke refers to a cerebral bleed due to a blood vessel's rupture (5). Ischemic stroke in turn comprises different subtypes such as transient ischemic attack (TIA), which is transitory and reversible in nature. We followed the classification system: Trial of Org 10172 in Acute Stroke Treatment (TOAST) developed by Adams et al. (6), and we further distinguished large-artery atherosclerosis, cardioembolic (CE), lacunar, undetermined etiology, and other determined etiology.

**Figure 1:**
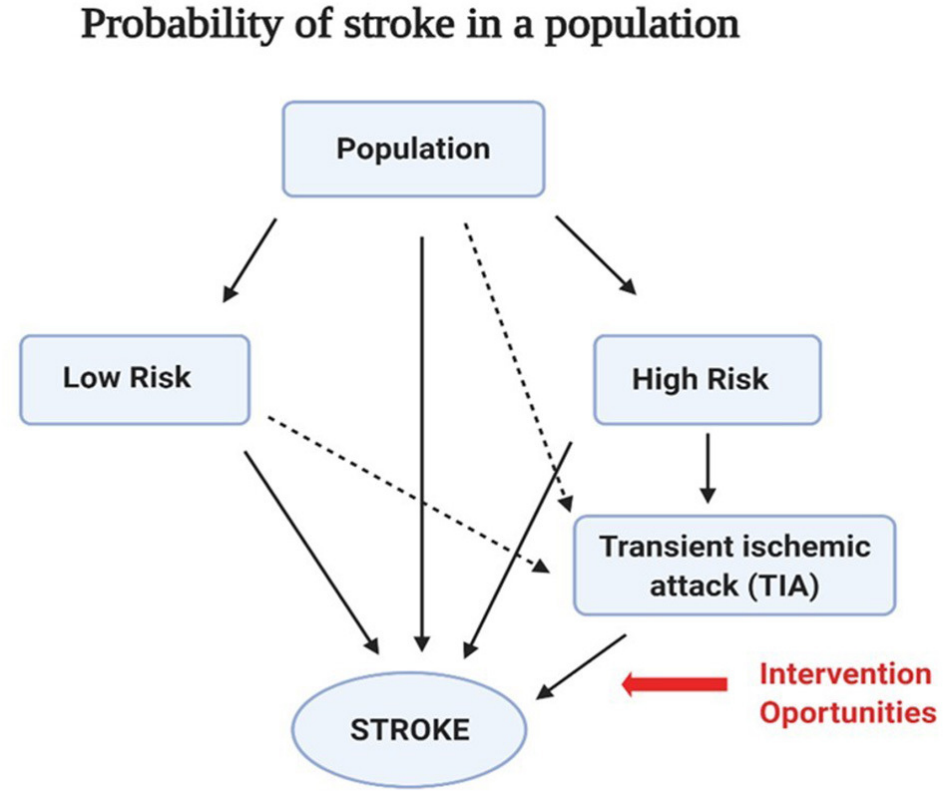
Changing risks of stroke recurrence in a population. Schematic diagram of the risk of stroke recurrence in the general population. There are mainly two groups: those with low risk (without any disease apparently) and those with high risk (people with prior cardiovascular diseases, diabetes, and hypertension). The probability of experiencing a transient ischemic attack (TIA) was higher in the high-risk group than that in the low-risk group. In addition, a TIA event increases the probability of recurrent stroke. Recognition of this risk structure provides a unique opportunity for early health interventions and stroke prevention.

Several studies have shown that subjects with TIA have a much higher probability of future strokes than the general population (7–9). In fact, the recurrence estimated by the Oxfordshire Community Stroke Project varies between 8 and 12% at 7 days, 11 and 15% at 1 month, and 15 and 19% at 3 months (8). Notably, recurrent events tend to become more disabling or fatal than the first stroke or TIA (9). Therefore, the first occurrence of TIA constitutes a warning signal for future stroke, offering a unique opportunity for early interventions and stroke prevention, including neuroprotective strategies (Figure 1). As one of the reviewers have highlighted, “acute stroke treatment is a time-critical process where every minute counts.”

Unfortunately, physicians may neglect these warning signals for recurrent future cerebrovascular events. In addition, misdiagnosis and untimely discharge are also relatively frequent (10). A TIA is a predictive factor for recurrence (11), and therefore, there is a strong need to determine the predictors of recurrence after the first TIA event. Early identification of patients at a higher risk for stroke recurrence may offer critical insights for urgent management and recurrence prevention. Stroke onset in patients requires additional factors that, ideally, are differentiation from stroke mimics, classification of stroke subtypes, and monitoring patient progression.

Early identification of such aspects is the goal of precision medicine for all diseases. This approach leverages disease progression models whose stages are identifiable using biomarkers (12, 13). In this framework, a biomarker is a parameter that may indicate the likelihood of disease progression or clinical events in subjects with a specific medical condition (14).

Regrettably, stroke remains to be a condition without well-established biomarkers, which, alone or in combination, are precise enough for a useful prediction. This situation seems contradictory, as an increasing number of biomarker candidates are continuously being proposed (15). However, selecting specific stroke biomarkers remains challenging for several reasons.

**Figure 2:**
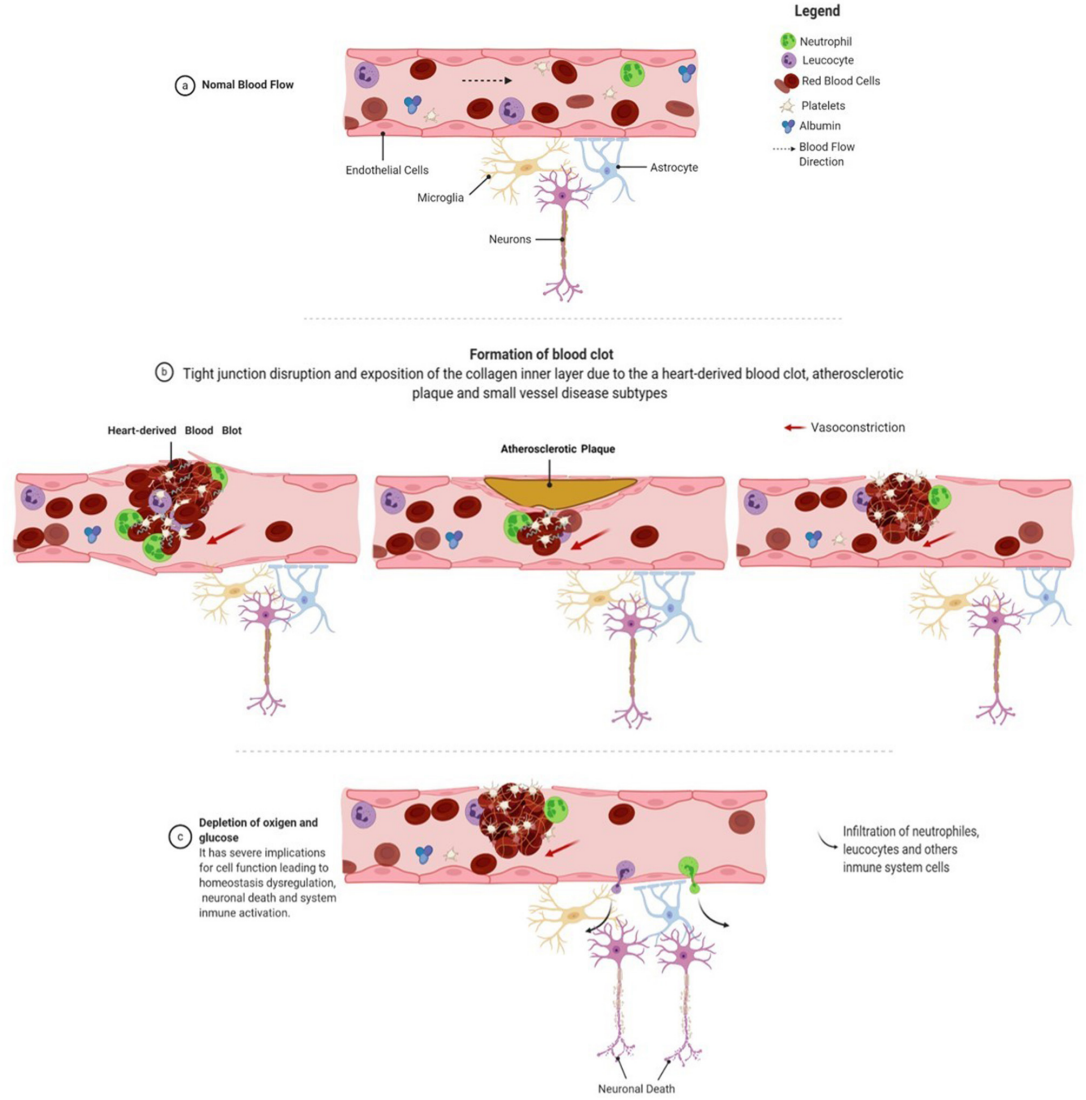
Stroke pathophysiology. The clot formation process starts with disrupting the blood–brain barrier due to a rupture of the endothelial cell layer, exposing the blood vessel's inner collagen layer. Collagen exposure is recognized by circulating platelets in the bloodstream, which initiates the process of aggregation. Fibrinogen is released from the liver to the bloodstream and is cleaved by thrombin at the damaged site, resulting in fibrin formation. Fibrin is one of the main constituents of blood clots, providing remarkable biochemical and mechanical stability. The blood clot is also composed of neutrophils and leukocytes that arrive at the injured site and form a solid structure that obstructs or reduces blood flow. The reduction in blood flow leads to a decrease in oxygen and glucose levels. These conditions favor a shift in the neuron's metabolic conditions, mitochondrial dysfunction, excitotoxicity, ion imbalance, and neuronal death. Figure was drawn using Biorender.com.

Stroke, as mentioned before, is a heterogeneous disease that involves diverse mechanisms that affect the specificity and sensitivity of potential biomarkers (16, 17). These mechanisms include disruption of the blood–brain barrier, thrombus formation, neuronal death, excitotoxicity, mitochondrial dysfunction, and immune system activation [(18); Figure 2]. Biomarkers may be sensitive to different facets of pathophysiology and may change over time.

Stroke diagnosis depends crucially on neuroimaging; computed tomography (CT) remains an essential component of stroke management, although it is not always available. Some areas of stroke management have been neglected, such as using biomarkers to predict stroke after TIA, and only a few studies have evaluated the risk of recurrent events in TIA subjects. Consequently, there is no clinical setting in which the use of a biomarker might help an individual patient. Acute stroke therapy is guided by the severity of the clinical symptoms and imaging. A preliminary study of the literature also indicated that single biomarkers achieved relatively low diagnostic accuracy.

To summarize, the use of biomarkers for stroke diagnosis is a challenging issue because, unlike for myocardial infarction, cerebral imaging remains the gold standard for stroke diagnosis. Therefore, expectations regarding the use of biomarkers in stroke patients should be realistic. We suggest that the primary use of biomarkers in stroke patients is to provide additional laboratory information to effectively distinguish between actual stroke and its mimics and to monitor patient condition and the effect of potential neuroprotective strategies.

To highlight promising directions, we present a systematic review of the literature on stroke biomarkers for the purposes mentioned above. This review comprises the following:

1. A preliminary review of the literature indicated that combinations of stroke biomarkers (“panels”) showed increased diagnostic accuracy. Thus, we focused on panels of biomarkers instead of isolated determinations.
2. We limited our attention to only those studies that reported the area under the receiver operating characteristic (ROC) curve (AUC). This choice allowed for quantitative comparisons of accuracy.
3. Because of the paucity of studies reported in the literature on subtype classification of stroke through biomarker combinations, we narrowed our search to the small-vessel-disease subtype of stroke.
4. We also focused our review on blood-based biomarkers as they seem to offer several advantages in terms of cost and ease of scalability (12).

As a consequence of our review, we propose new combinations that highlight the pathophysiological processes related to the selected biomarkers.

Overall, we adopted this scope for our review because of the geographical distribution of stroke. The highest incidence of stroke has been reported in high-income countries. Better reporting and shifting demographics place the onus on the developing world, with an increase of 91.4 million disability-adjusted life-years and 4.85 million deaths in proportion to all global causes (4, 19). Thus, technologies that are deployable without advanced analytical or imaging technologies need to be explored in more detail. Blood-based biomarker panels may therefore contribute in providing valuable information for the management of stroke.

## Methods

### Article Search

We developed a search strategy with assistance from a research committee formed by neurologists, molecular biologists, mathematicians, and bioinformaticians. The search strategy was established using a combination of standardized MeSH (Medical Subject Headings) terms and keywords, including but not limited to (-cerebrovascular disorder or brain vascular disorders or vascular diseases, intracranial or intracranial vascular disease or cerebrovascular occlusion or cerebrovascular accident or intracranial embolism, and thrombosis or cerebrovascular insufficiencies) **AND** (- ischemia or Stroke or infarction or brain infarction or hypoxia-ischemia or brain ischemia or ischemic attack) **AND** (-intracerebral hemorrhage, cerebral hemorrhage, or intracranial hemorrhage) **AND** (- biological marker or biomarker or biologic marker or marker, biological, or biomarker panel) **AND** (- blood plasma sample, serum plasma sample, cerebrospinal fluid, blood proteins, plasma, blood, marker, serum, or serum marker or laboratory markers) **AND** (- diagnoses or diagnostic or examinations). The search encompassed studies conducted between 1966 and June 2020 for studies in patients with suspected stroke; the inclusion and exclusion criteria are provided below. The PubMed search was conducted on October 10, 2020, at 12:48:21 P.M.

### Eligibility Criteria

**Figure 3:**
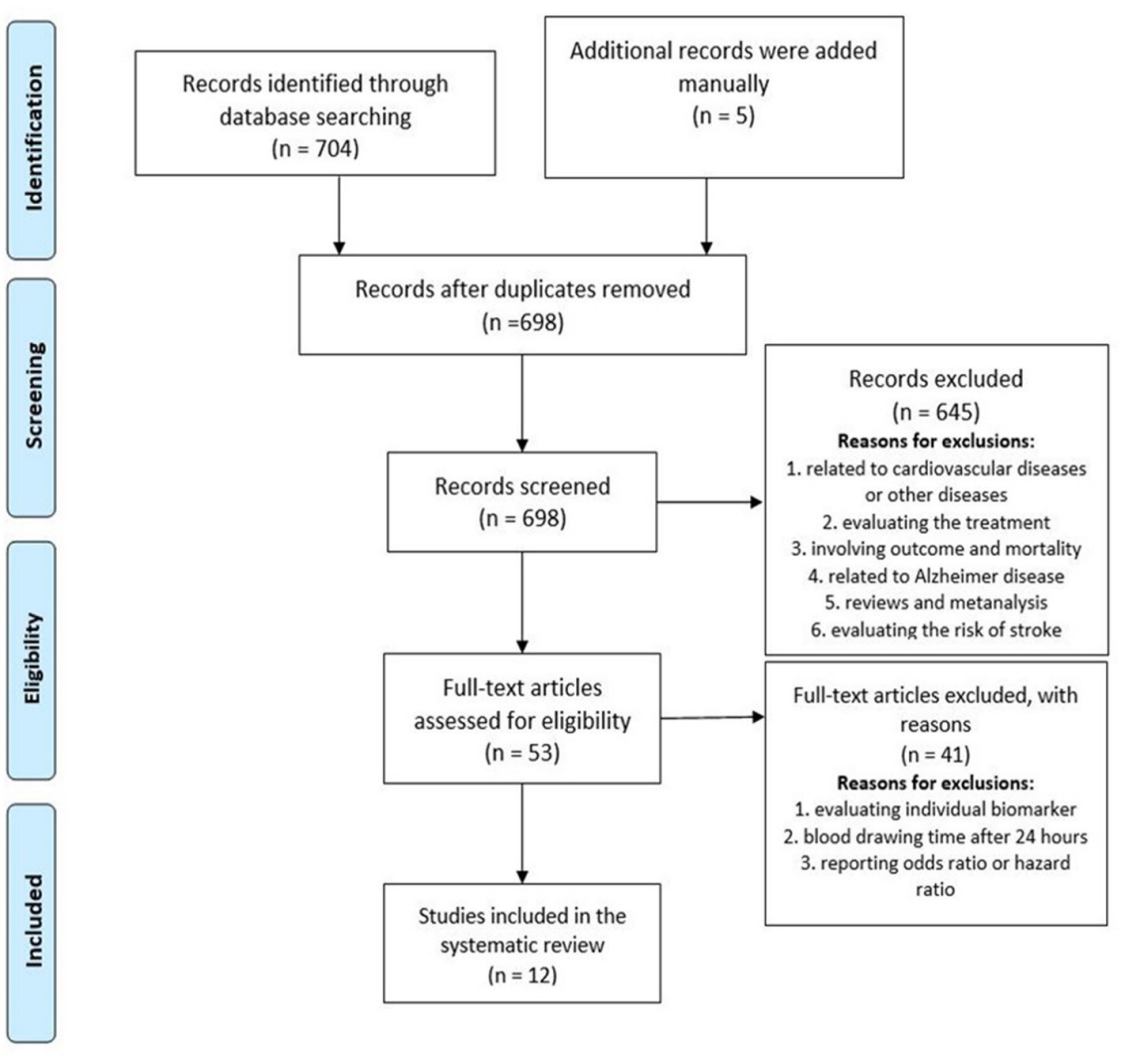
PRISMA diagram. Flow diagram of the search and screening process. PubMed research yielded 704 articles at baseline, and five were added manually. No other sources of article identification were identified.

The studies that were included met the following criteria: (1) case–control studies; (2) patients aged ≥18 years; (3) magnetic resonance imaging or CT performed to confirm the clinical diagnosis of ischemic stroke; (4) a blood or serum biomarker assessed within 0–24 h after symptom onset; (5) the study reported the relationship between biomarker level and diagnostic accuracy; (6) the study included two or more biomarkers because the use of a biomarker panel improved the sensitivity and specificity for identifying cases of stroke in comparison with a single biomarker (20); and (7) the study reported the sensitivity, specificity, and AUC for the model for stroke diagnosis. We selected articles written in English or Spanish. Reviews, conference abstracts, and editorial letters were excluded. Mendeley was the reference management software used for the identification, elimination of duplicates, and screening purposes. The studies were selected based on the title and abstract for one author in the first phase (SB). In the second phase, we read the full text of the preselected articles and included studies matching the eligibility criteria (Figure 3). Disagreements were resolved by consensus.

### Data Extraction

One of the authors (SB) extracted all the data needed to meet the review goals, including publication year, first author, sample size (*n*), biomarkers used, assays used to measure the biomarker, biomarker cutoff value used (if available), blood draw time, and the values of AUC, sensitivity, and specificity. This review followed the Preferred Reporting Items for Systematic Reviews and Meta-Analysis (PRISMA) guidelines for a systematic review (21).

## Results

### Search Results

A total of 704 articles emerged from the initial search process as potentially relevant records, and five were added manually based on the author's recommendations; 698 studies remained after the manual removal of duplicates. The screening process ruled out 645 articles based on abstract and title for the following reasons: articles related to cardiovascular diseases or other diseases (pulmonary embolism, Alzheimer disease, renal disorders, and others); articles evaluating the risk of stroke; and reviews and meta-analyses involving outcome and mortality and being related to genetic biomarkers. Finally, 53 full reads were selected and assessed for eligibility, and 41 were eliminated because of evaluating individual biomarkers, drawing blood after 24 h, and not reporting AUC, sensitivity, or specificity. Finally, 12 articles were included in the systematic review. A PRISMA flow diagram describing the search and screening process is shown in Figure 3.

An example of a systematic review that was not included in our evaluation because of a lack of statistical measures of diagnostic accuracy was a recent meta-analysis evaluating several biomarkers (25). Most of the biomarkers evaluated in this study are reported in the literature and are reviewed as potential candidates and are added in several panels below; however, von Willebrand factor (vWF) and NR2 were omitted. Note that glial fibrillary acidic protein (GFAP) was the most promising biomarker in the study of separate ischemic stroke (IS), intracerebral hemorrhage (ICH), and healthy controls. The same study mentions d-dimer, matrix metalloproteinase 9 (MMP-9), brain natriuretic peptide (BNP), and protein S100-β (S100β) derived from the meta-regression analysis as significant markers to be evaluated within 6 and 24 h of symptom onset (22).

### Study Characteristics

The main features of the selected studies are listed in Table 1. Regarding sample characteristics, all the studies were case–control, which included control participants (without stroke), patients with acute IS (AIS), ICH, TIA, mimics, closed-head injuries (CLHs), or subarachnoid hemorrhage (SAH). All the studies involved subjects aged ≥18 years, and in the majority, immunoassays were used to evaluate the levels of biomarkers. Most of them reported their values of sensitivity, specificity, or AUC obtained within the first 6 h of symptom onset using multivariate or univariate regression logistic analyses.

**Table 1:** Summary of the 12 articles included in the systematic review.

**Biomarker panel**	**Sample size (*n*)/groups**	**Time**	**Essays**	**Cut off**	**Specificity**	**Sensitivity**	**Area under the curve**	**References**
BNP, D-dimer, MMP-9, and S100β ICH vs. mimics	*n* = 946 subjects 293 (IS), 95 (ICH), 197 (TIA), and 361 (mimics) 343 subjects for the validation cohort: 87 (IS), 64 (ICH), 40 (TIA), and 152 (mimics) **ICH vs. mimics**	0–3 h 3–6 h	Triage Stroke Panel (fluorescence immunoassay)	MMP-9: 25–1,300 ng/mL D-dimer: 150–5,000 ng/mL S100β: 100–8,000 pg/mL BNP: 10–5,000 pg/mL	Stroke vs. mimics: 45% ICH vs. mimics: 38%	Stroke vs. mimics: 90% ICH vs. mimics: 88%	Stroke vs. mimics: 0.75 ICH vs. mimics: 0.81	(23)
BNP, D-dimer, MMP-9, S100β, and multimarker index (MMX)	*n* = 139 subjects 89 (AIS), 11 (ICH), and 39 (brain disorders) **AIS vs. others (control, brain diseases)**	<6 h	Triage® Meter (Biosite Inc.) fluorescence immunoassay	MMP-9: 25–1,300 ng/mL D-dimer: 150–5,000 ng/mL S100β: 100–8,000 pg/mL BNP: 10–5,000 pg/mL	21.5%	91.0%	0.714	(24)
BNP, D-dimer, MMP-9, S100β, and multimarker index (MMX)	*n* = 174 subjects 100 (IS), 49 (mimics), and 25 (TIA) **IS vs. mimics**	<6 h	Triage® Stroke Panel	—	33%	86%	0.59	(25)
Eotaxin, EGFR, S100A12, TIMP-4, and prolactin	*n* = 167 subjects 57 (IS), 32 (ICH), 41 (TIA), and 37 (mimics) **IS + ICH vs. mimics**	<24 h	—	—	84% IS vs. mimics (*c* = 0.92)	90%	0.97	(26)
D-dimer, caspase-3, sRAGE, MMP-9 chimerin, and secretagogin	*n* = 1,005 subjects 776 (IS), 139 (ICH), and 90 (mimics) **IS vs. mimics**	<6 h	ELISA immunoassays	Caspase-3: 1.962 ng/mL D-dimer: 0.275 μg/mL sRAGE: 0.91 ng/mL chimerin: 1.11 ng/mL secretagogin: 0.24 ng/mL MMP-9: 199 ng/mL	63%	84%	0.810 (0.757–0.863)	(27)
S100β, MMP-9, VCAM, and vWF	*n* = 80 subjects 44 (IS), 21 (controls), 13 (TIA), 1 (syncope), 1 other conditions **Stroke vs. controls**	<6 h 6–24 h	Biosite Inc.	—	90%	90%	—	(28)
S100β, BNGF, vWF, MMP-9, and MCP-1	*n* = 274 subjects 82 (ISCH), 65 (SAH), 38 (ICH), 38 (closed head injuries, CLH), and 51 (TIA) **AIS vs. other groups** **ICH vs.other groups**	<6 h	ELISA-immunoassays	—	93%	AIS: 91.7% ICH: 80%	—	(29)
IMA index IMA	*n* = 52 subjects 28 (IS) and 24 (No-Stroke) **IS vs. no stroke**	<6 h	Albumin–cobalt binding (ACB) test	91.4 U/mL	96.4%	95.8%	0.990 (0.970–1.000)	(30)
AT-III Fibrinogen	*n* = 198 subjects 152 (IS) and 46 (mimics) **IS vs. no stroke**	4.5 h	AT-III: chromogenic assay Fib: immunoturbidimetry assay	AT-III: 210% Fib: 4 g/L	AT-III:93.62% F: 82.61%	AT-III: 97.37% F: 96.05%	—	(31)
Ab NR2 and GFAP	*n* = 124 subjects 49 (IS), 23 (ICH), 52 controls **IS vs. ICH**	<12 h	GFAP: ELISA kit AB NR2: Gold Dot NR2 Antibody Assay kit (ELISA)	—	91%	94%	—	(32)
GFAP/UCH-L1 GFAP	*n* = 184 subjects 45 (ICH), 79 (IS), 5 (SAH), 3 (TIA), and 57 controls **IS vs. controls** **ICH vs. controls** **IS vs. ICH**	<4.5 h	ELISA immunoassays	GFAP: 0.34 ng/mL	— —	— —	0.875 0.71 **(IS vs. C)** 0.95 **(ICH vs. C)** 0.86 **(IS vs. ICH)**	(33)
ApoC-III, NT-proBNP, and FasL (21 biomarkers)	*n* = 1,308 subjects 941 (IS), 193 (mimics) and 174 (hemorrhagic) 767 validation cohort **IS vs. mimics** **IS vs. ICH**	<6 h	ELISA immunoassays	—	—	—	**IS vs. mimics** 0.742 (0.686–0.797) **IS vs. ICH** 0.757 (0.691–0.823)	(34)

*ROC analysis includes clinical variables selected by the authors. Bold values indicate groups compared in the studies analyzed by Receiver Operating Curve analysis (ROC)*.

### Biomarker Analysis Based on Selected Studies

One of the biomarker panels frequently evaluated for the identification of AIS is composed of four proteins: BNP, d-dimer, S100β, and MMP-9 (23–25). The results of previous studies have been mixed as follows:

- Laskowitz et al. (23) showed that combining these four proteins outperformed other biomarkers in differentiating mimics from intracranial hemorrhage cases, with *c* = 0.76. This result was validated in a study of 293 subjects, 361 mimics, and 197 TIA with a validation cohort of 343 suspected stroke cases. The study's global results to classify stroke cases exhibited a high sensitivity of ~90% but a low specificity of ~45%, and 91% sensitivity and 45% specificity for differentiating specific IS.
- Kim et al. (24) validated the previous biomarker panel using the composite multimarker index (MMX), which combines the individual marker values into a single index value. It exhibited good performance in the discrimination of patients with acute infarction; at 6 h, it could differentiate AIS (*p* < 0.001) but with insufficient precision. With 91.0% sensitivity, 21.3% specificity, and 71.4% AUC, the analysis exhibited a modest discriminatory power for acute stroke. According to the MMX values, there was no significant difference between the subjects with AIS and those with ICH (*p* = 0.884).
- These promising results should be considered with caution. Knauer et al. (25) evaluated MMX in a cohort of 174 cases in which 100 patients had stroke, 49 were mimics, and 25 had TIA. They advised against the use of the panel BNP, d-dimer, S100β, and MMP-9 in this assay because of (1) the low significance of MMX values to differentiate the IS group (MMX = 3.6 ± 2.0) from mimics (MMX = 4.2 ± 1.7) and (2) the low significance of MMX values when the analysis for the individual biomarkers was replicated. The 2.3 cutoff value of MMX was reported to have 86% sensitivity with a low specificity of ~33% and an AUC of ~59%.

A panel comprising eotaxin, EGFR, S100A12, metalloproteinase inhibitor-4 (TIMP-4), and prolactin was found to be elevated in a study of 167 cases with neurologic deficits, allowing the differentiation of IS cases from mimics (*c* = 0.92) (26). The study used a time window of 24 h and reported a high specificity of ~84%, sensitivity of ~90%, and AUC of ~97%.

Another panel, caspase-3, d-dimer, chimerin-II, MMP-9, secretagogin, and sRAGE, was assessed in a large cohort of 1,005 cases where 915 had strokes and 90 had stroke-mimicking conditions, but only proteins could discriminate between the two groups (27). At 6 h after symptom onset, these protein levels had moderate sensitivity values of ~84% but a low specificity of ~63%.

The panel S100β, MMP-9, vascular cell adhesion molecule (VCAM), and vWF proved to have good discriminatory power as it differentiated 44 AIS cases from 21 controls within the first 6 h after symptom onset with high sensitivity (~90%) and specificity (~90%).

A panel slightly modified from the previous one comprising S100β, MMP-9, and vWF with two other markers including monocyte chemoattractant protein-1 (MCP-1) and B-type neurotrophic growth factor (BNGF) had similar discriminatory power (29). The levels for the panel in samples of 82 ISCH (IS with ICH), 65 SAH, 38 ICH, 38 CLH, and 51 TIA patients, at 6 h from symptom onset and using multivariate logistic regression model, had elevated sensitivity of ~92% and specificity of ~93% in the classification of ischemic events and a specificity of ~93% and sensitivity of ~80% for the prediction of hemorrhagic stroke.

The protein GFAP has been of interest in combination with other biomarkers. The combination of antibodies (Ab) against NR2 and GFAP exhibited the best predictive power for comparing 49 IS subjects from 23 ICH patients and 52 controls. A sensitivity of ~91% and specificity of ~94% were reported within 12 h of symptom onset (32). The use of GFAP and UCH-L1 for the identification of ICH vs. IS was tested in 129 stroke subjects, three TIA patients, and 57 controls (33). Notably, GFAP alone was capable of distinguishing between the condition with an AUC ~0.86, sensitivity of 61%, and specificity of 96% (33).

Recently, a panel consisting of apolipoprotein CIII (Apo C-III), NT-proBNP, and FasL was selected as the best combination after an extensive study of 21 biomarkers in 1,308 cases to differentiate real stroke from mimics, within 6 h after stroke onset (34). This study was replicated with a smaller sample size for a different group of subjects, giving a modest accuracy of 0.742 (0.686–0.797). Despite being one of the studies that screened the largest number of biomarkers, this, in our opinion, has some limitations. GFAP was not assayed, and the levels of MMP-9 were not measured in the entire cohort because they were not deemed discriminative (34).

Finally, it is worth mentioning the two articles that screened several biomarkers, although they did not combine them. They can be integrated into an optimized panel in the future. The studies and biomarkers are as follows:

- Ischemia-modified albumin (IMA): in a small number of patients (*n* = 28) with stroke compared to the no-stroke group (*n* = 24) where the albumin-adjusted IMA index and IMA were measured within 6 h after symptom onset (30). Furthermore, the IMA index (98 U/mL) was even more sensitive (sensitivity, ~95.8%; specificity, ~96.4%; and AUC, ~99%) than the conventional IMA value (sensitivity, ~87.5%; specificity, 89.3%; and AUC, 92.8%) for the detection of patients with cerebral ischemia.
- The levels of antithrombin III (AT-III) in a study with 152 stroke patients and 46 mimics reported the highest sensitivity of ~97.37% and specificity of ~93.62% using a cutoff of 210%, whereas 4 g/L of fibrinogen reached a sensitivity of ~96.05% with a specificity of ~82.61% (31).

### Observations From the Selected Studies

1. Only two biomarkers, NR2 peptide (Ab against NR2 fragment) and GFAP, have been reported as brain-specific markers linked to the progression of stroke, reaching the highest predictive power when evaluated together (32).
2. Two proteins that have been widely used as biomarkers are vWF and MMP-9; however, they are not specific to stroke. Although they were evaluated in a combined panel with higher accuracy (28, 29), Reynolds et al. reported that vWF and MMP-9 alone could not be used for diagnosis. However, it has good univariate discrimination of non-diseased vs. diseased, with an added discriminatory capacity to the logistic regression model [*p* < 0.0001; (29)].
3. S100β is one of the most evaluated biomarkers; however, it is also not specific to stroke. Along with GFAP, it is one of the strong candidates for the differentiation of hemorrhagic and ischemic subtypes in the acute phase of stroke (35).
4. An IMA index is the IMA value multiplied by individual serum albumin concentration/median albumin of the study population (36). The IMA index seems to be more specific than IMA in the differentiation of IS, but it has not been used in combination in previous studies.
5. According to previous studies, the use of AT-III and fibrinogen might help in distinguishing between conditions with high accuracy in individual analyses (31).
6. Usually, ROC curves are the statistical method to compare two groups of patients: ICH vs. mimics, AIS vs. other groups, IS vs. mimics, and IS + ICH vs. mimics.
7. Note that none of these results have been approved for advanced clinical trials.

## Proteins Derived From the Systematic Search and Their Relation to the Pathophysiology of Stroke

**Figure 4:**
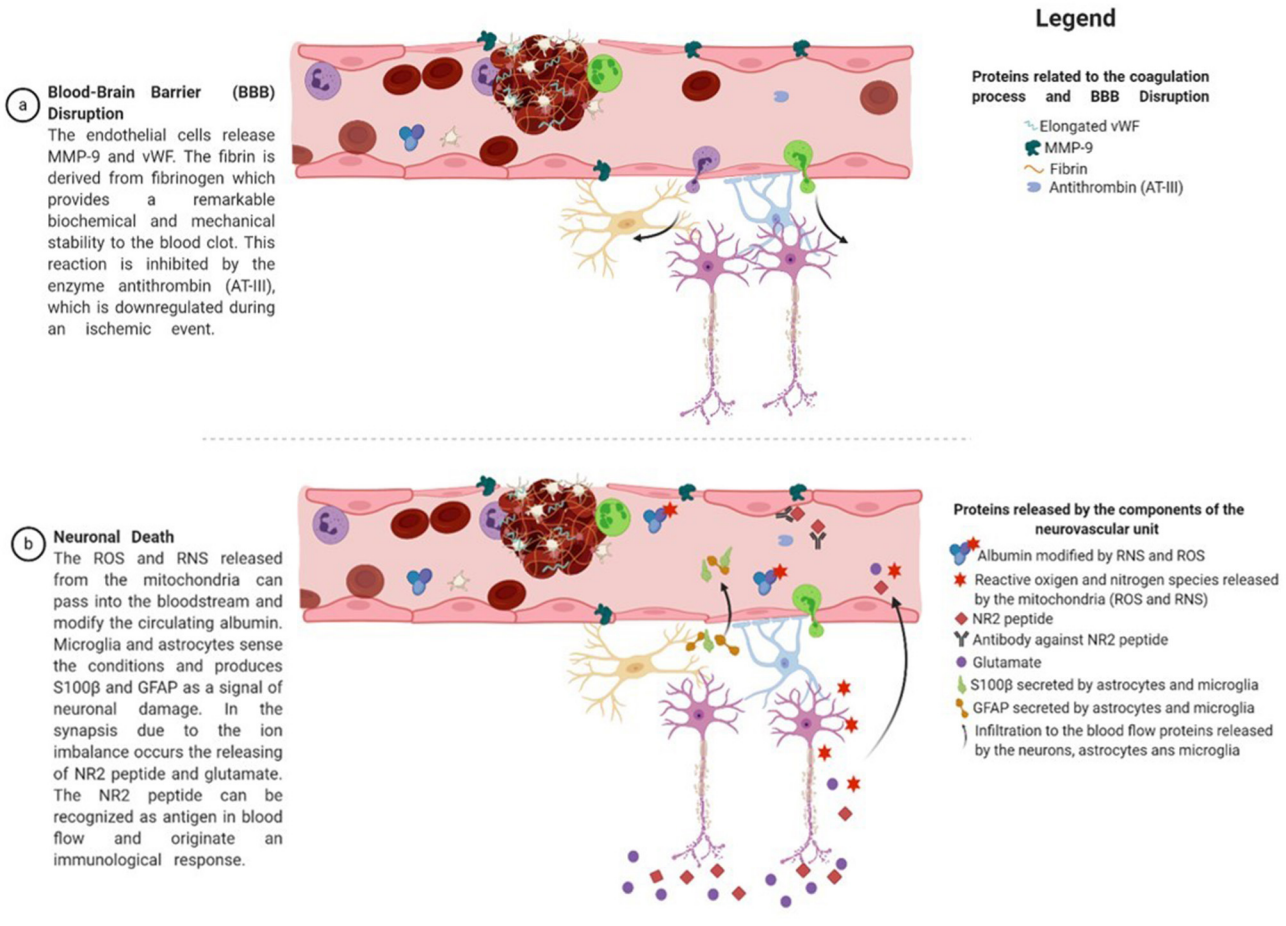
Proteins derived from the proposed panel of biomarkers and their relation to the pathophysiology of stroke. After an injury, endothelial cells release MMP-9, which plays an essential role in the extracellular matrix and local proteolysis of leukocyte migration. In addition, endothelial cells release vWF in a globular form, which is then transformed into an elongated form by the activity of the ADAMTS13 enzyme. vWF promotes platelet adhesion to the damaged site by forming a molecular bridge between the subendothelial collagen matrix and the platelet-surface receptor complex GPIb-IX-V. Fibrinogen is released from the liver to the bloodstream and is cleaved by thrombin at the damaged site, resulting in fibrin formation. Fibrin is one of the main constituents of blood clots and provides remarkable biochemical and mechanical stability. The conversion of fibrinogen to fibrin by thrombin is inhibited by the enzyme antithrombin (AT-III), which is downregulated during an ischemic event. In addition, neutrophils and leukocytes arrive at the injured site and join with the previous proteins to form a solid structure that obstructs or reduces blood flow. These conditions lead to a shift in the neuron's metabolic conditions, mitochondrial dysfunction, excitotoxicity, and ion imbalance. The reactive oxygen and nitrogen species released from the mitochondria can pass into the bloodstream and modify the circulating albumin. On the other hand, microglia and astrocytes sense the conditions and produce S100β and GFAP to signal neuronal damage. In synapsis, because of ion imbalance, the release of the NR2 peptide and glutamate occurs. The NR2 peptide can be recognized as an antigen in the blood flow and induces an immunological response. Figure was drawn using Biorender.com.

We have selectively outlined concepts of stroke pathophysiology that justified our proposals for novel biomarker panels for clinical prediction explained with graphical details in Figure 4:

1. When rupture of the endothelial layer of the vessel occurs, the inner collagen layer is exposed. The exposure of collagen with blood is recognized by platelets, forming a sticky plug that initiates clot formation (37). Consequently, endothelial cells release vWF, MMP-9, P-selectin, E-selectin, and inflammatory mediators (38). vWF promotes platelet adhesion to the damaged site by forming a molecular bridge between the subendothelial collagen matrix and the platelet-surface receptor complex GPIb-IX-V (39). Fibrinogen is released from the liver to the bloodstream and is cleaved by thrombin at the damaged site, resulting in fibrin formation. Fibrin is one of the main constituents of blood clots and provides remarkable biochemical and mechanical stability (40). The conversion of fibrinogen to fibrin by thrombin is inhibited by the enzyme AT-III, which is downregulated during an ischemic event. Neutrophils and leukocytes also adhere to the injury site and form a solid structure that obstructs or reduces blood (Figure 2).
2. Flow reduction leads to the depletion of oxygen and glucose, which has severe implications for cell function, resulting in dysregulation of neuronal homeostasis. The process of intracellular medium acidification is reported when metabolism changes to anaerobic conditions (41). Mitochondrial dysfunction plays a significant role in inducing neuronal death caused by an increase in enzymes that favor the production of reactive oxygen species (ROS) and reactive nitrogen species (RNS), such as xanthine oxidase, NADPH oxidases, nitric oxide synthase, and a decrease in detoxifying systems (42, 43). Note that ROS and RNS pass into the bloodstream and are hypothesized to modify the N-terminal of albumin (44).
3. Mitochondrial dysfunction affects ATP production, which induces a failure in the activity of the Na^+^–K^+^ pump. Na^+^–K^+^ pump activity depends on ATP hydrolysis (45), disappearing the electrical gradient in the cellular membrane and causing the influx of Na^2+^ into the neuron, resulting in membrane depolarization. Simultaneously, the activation of ASC1a, NCX, and TRP allows the influx of Ca^2+^ into the neuron, a process known as calcium overloading (46). Calcium overloading favors glutamate release into the extracellular medium and causes swelling due to the influx of water. NR2 is a subunit of the *N*-methyl-d-aspartate (NMDA) receptor and is a ligand-gated ion channel with high calcium permeability, which is cleaved by serine proteases under ischemic conditions (47).
4. All these processes lead to neuronal death by necrosis or necroptosis, characterized by the loss of membrane integrity, damage to cellular structures, swelling, and release of cellular content, resulting in an acute inflammatory response (48). Consequently, there is an activation of astrocytes and microglia, which induces morphological changes and mediates the inflammatory response (49–51). These cells release proteins such as S100β and GFAP, reflecting structural and functional damage in the central nervous system (CNS) (35). MMP-9 is released by endothelial cells, astrocytes, and microglia and is activated by high nitric oxide concentrations. It degrades type IV collagen present in the endothelial blood–brain barrier, increasing parenchymal destruction, and is related to the inflammatory response after stroke (52).

## Proposal for New Biomarker Panels

Based on our reviewed pathophysiology, we suggest further studies of three different panels (Table 2). We have included NR2 peptide and GFAP in all proposed panels because they seem to be the most promising brain-specific biomarkers related to stroke. We have included other biomarkers in the suggested panels under *Observations From the Selected Studies* described above because they seem promising in light of the pathophysiological process of stroke that have been previously evaluated (Table 3).

**Table 2:** Proposal of biomarker panels for stroke recurrence.

**Biomarker panel proposal**
NR2+ GFAP+ MMP-9 + vWF + S100β
NR2 + GFAP + MMP-9 + vWF +IMA index
NR2+ GFAP+ AT-III + fibrinogen

**Table 3:** Biomarkers derived from the biomarker panel proposal and their possible role identified in stroke.

**Proteins**	**Gene name**	**Protein name**	**Description**	**Functions**	**References**
NR2 peptide	GRIN1 GRIN2A GRIN2B GRIN2C	Glutamate receptor ionotropic, NMDA 2A, and NMDA 2B	It is a ligand-gated ion channel with high calcium permeability and voltage-dependent sensitivity to magnesium. It is essential in the process of neuronal synapses.	As a response to the brain's ischemic conditions, serine proteases are activated, which causes the cleavage of the NR2 subunit of NMDA receptors (NMDARs). Then its subunit is released to the blood, being a marker of neuronal damage.	(47)
GFAP	GFAP	Glial fibrillary acidic protein	It is an intermediate filament class-III. It is classified as a glial marker.	After an injury, trauma, disease, genetic disorders, or chemical insult GFAP is released from reactive astrocytes. The process is named astrogliosis and reflects structural and functional damage in the CNS.	(51, 53)
S100β	S100β	Protein S100-β	It is a protein-related to calcium metabolism. Also, it participates in the transmission of intracellular signals through second messengers. It is involved in the development and maintenance of the CNS. It is classified as a glial marker.	It is released from astrocytes and microglia after an injury or trauma, reflecting the CNS's structural and functional damage. It is directly related to the volume of lesions, clinical status, and functional outcome.	(29, 50)
vWF	VWF	von Willebrand factor	It plays an essential role in the maintenance of hemostasis, blood coagulation, and cell adhesion.	It is secreted by the endothelial cell activated in response to injury and can adhere to circulating platelets and contributing to thrombus formation.	(54)
MMP-9	MMP9	Matrix metallopeptidase 9	It is a proteolytic enzyme belonging to the group of gelatinases. It has an essential role in local proteolysis of the extracellular matrix and leukocyte migration.	It is activated by high concentrations of oxide nitric and degrades the type IV collagen present in the endothelial blood–brain barrier, increasing its parenchymal destruction. It is related to the inflammatory response after stroke.	(52, 55)
IMA index (albumin)	ALB	Albumin	It is the major transporter of Zn, Ca^2+^, Mg in plasma and binds water Na^+^, K^+^, fatty acids, hormones, etc. It regulates the colloidal osmotic pressure of blood.	Under ischemic conditions, it is a measure of oxidative stress, where the NH_2_ terminus of human albumin may be modified for the free radicals, but the precise mechanism is yet unknown.	(30, 44, 56)
AT-III	SERPINC1	Antithrombin III	It is a plasma serine protease inhibitor that regulates the blood coagulation cascade and inhibits the thrombin.	Inactive AT-III–thrombin complexes are formed during the acute phase of stroke.	(57, 58)
Fibrinogen	FGA, FGB, FGG	Fibrinogen	It is a blood glycoprotein essential in coagulation and determines the plasma viscosity.	It is related to the thrombosis process favoring the platelet aggregation after injury, being one of the primary components of blood clots.	(40, 59)

Panel 1: NR2 + GFAP + MMP-9 + vWF + S100β

- **NR2**: Precisely, the NR2 subunit is the only biomarker reported with the highest specificity (96%) and sensitivity (92%) at 12 h using a cutoff value of 1.0 μg/L in 101 IS and 91 no-stroke patients (60). When a lower cutoff value of 0.5 ng/mL was tested within 0.5–4.5 h, it revealed high sensitivity and specificity of 88 and 99%, respectively, for the differentiation between mild traumatic brain injury, AIS, ICH, healthy controls, and subjects at risk of TIA (vascular risk factors) (https://www.ahajournals.org/doi/10.1161/str.44.suppl_1.A30). Additionally, the concentrations of NR2 were found to be significantly elevated in IS subjects compared with patients without cerebral damage and were also related to the size of infarct and were used as a blood test for the validation of Cortexin treatment, a neurocytoprotector (61).
- **GFAP**: The predictive value of GFAP to determine the types of stroke was assessed by Foerch, who used a cutoff value of 2.9 ng/L, obtaining a sensitivity of 79% and a specificity of 98% (53). The same author, after several years, ratified GFAP as an efficient marker to differentiate ICH from IS, including stroke mimics [AUC = ~0.915; 95% confidence interval (CI) = 0.847–0.982; *p* < 0.001; (62)]. Recently, the potential of using a value of 0.43 ng/mL was found, achieving the highest diagnostic accuracy for the differentiation between ICH and AIS (sensitivity ~91%, specificity ~97%) within 6 h after symptom onset (63). Ren et al. demonstrated that the median concentration of this protein in patients with IS with no history of stroke was lower than that in cases with a history of stroke (0.015 vs. 0.07 ng/mL, respectively, *p* = 0.004) (33).
- **vWF** was found to be higher in stroke patients, and it has been associated with the CE and large-vessel disease (LVD) subtypes (64). Additionally, its levels have been related to the severity of arterial thrombus formation and poor functional outcomes (65, 66). In a proteomics prospective clinical study, vWF could differentiate TIA and minor stroke from non-cerebrovascular (mimic) conditions [1.256 (1.034–1.527)] (67). Furthermore, vWF can be a sign of the response following thrombolytic therapy or endovascular treatment in IS patients (65, 68).
- **MMP-9**: Higher concentrations of this protein have been observed early, in the acute phase, and later in stroke (69). MMP-9 levels are correlated with infarct volume, neurological deficits, and infarct progression (70, 71). This could be a measure of the transformation to hemorrhagic stroke after thrombolytic treatment (69, 72, 73). MMP-9 levels may be indicative of an inflammatory response after stroke (55), endothelial dysfunction (52), and response to thrombolytic treatment after IS (69, 72, 74). Recently, Misra et al. concluded that its levels could differentiate between IS, ICH, stroke mimics, and control subjects (*p* < 0.05) in a systematic review and meta-analysis of studies realized at 24 h. However, within 6 h, IS could not be differentiated from other groups (ICH, stroke mimics, and controls) (22).
- **S100β** is released early into the peripheral blood and is correlated with the National Institutes of Health Stroke Scale scores, infarct volume, and severity (29, 75). Foerch et al. reported that it could be used for <6 h as an indirect time and for successful clot lysis (75). At days 2 to 4 after acute stroke, S100β can be predictive of the disease's course with higher accuracy, associating the higher levels with the worst functional status, making its evaluation an effective recurrent biomarker (76, 77). S100β was able to separate IS from mimics [standardized mean difference (SMD) = 0.41; 95% CI = 0.18–0.63] but failed to identify healthy controls, as well as ICH at 6 h (22). In addition, no significant differences were reported in an analysis that compared biomarker values between different conditions (34).

Panel 2: NR2 + GFAP + MMP-9 + vWF+ IMA index

We have decided to eliminate S100β in the second and third panels because GFAP is a much more brain-specific biomarker (35). We have added the IMA index in this panel because it has not been previously assayed.

- **IMA index:** Several studies have probed the use of IMA as a diagnostic marker of acute coronary syndrome and AIS (30, 56, 78). Their admission levels were also associated with people suffering from acute aortic dissection (79) and can differentiate between ICH and IS patients (80).

Panel 3: NR2 + GFAP+ AT-III + fibrinogen

We have decided to eliminate MMP-9 and vWF in the third panel because AT-III and fibrinogen are also biomarkers of the coagulation process and reflect the thrombotic status. Both proteins were individually evaluated in a meta-analysis, which did not show significant differences (22), but we recommend their assessment together in a multivariate regression logistic model.

- **Fibrinogen** is one of the markers with an essential role in the thrombosis process because it is related to platelet aggregation after injury and inflammation (40, 59). In the case of an ischemic event, an association between elevated levels of this protein and increased risk has been reported (81, 82). Fibrinogen has been used to evaluate the long-term outcome and the size of the clot burden in patients after stroke (83, 84).
- **AT-III** is involved in the blood coagulation cascade, and inactive AT-III-thrombin complexes are formed during the acute phase of stroke (57). Peripheral blood concentrations are correlated with infarct severity and predict clinical outcomes and recurrence (58).

## Discussion

### Main Findings

To the best of our knowledge, this is the first systematic and comprehensive study to summarize current evidence regarding the use of combinations of biomarkers in the early stages of stroke. A sobering observation is that, despite numerous published studies, none of the protein biomarkers reported, alone or in combination, have been approved for the clinical management of stroke. It seems that, at best, these biomarkers can serve as support for clinical and imaging evaluation of patients. The objective of this study was to facilitate the identification of stroke mimics and allow dynamic follow-up of a patient's state to guide neuroprotective interventions. The diagnostic accuracy of stroke biomarkers must be accurate and time-sensitive to allow such dynamic follow-up. A significant result from our review is that combinations of biomarkers exhibit higher diagnostic accuracy than isolated biomarkers. Thus, there seems to be a substantial area for improvement by employing biomarker panels.

Based on our review, we suggest using three new panels of protein biomarkers to evaluate the pathophysiology of stroke. We have noted that the combination of GFAP and NR2 is included to determine neuronal damage with high accuracy in all three proposed panels. NR2 peptide is a brain-specific biomarker that has shown promising results for the distinguishing stroke, with one of the highest reported accuracies. Therefore, it is surprising that few studies have used it in combination with other proteins. The suggestion to include it in all three panels is based merely on this fact.

An intriguing possibility is that we might be able to monitor people with hemorrhagic and IS using a combination of S100β, GFAP, and IMA indices. S100β and GFAP have the same kinetics during cerebrovascular events. During ICH, both proteins peak early, before 24 h. The peak was reached later for the ischemic events. This difference in kinetics suggests that early peaking of blood levels of S100β and GFAP could be the hallmark of ICH during the acute phase of stroke (35). This crucial, yet still unresolved, distinction between IS and ICH is essential to make a decision about interventions. Likewise, blood proteins can contribute to treatment optimization for ISs by providing detailed information about hemostatic conditions involving pathways of coagulation activation, fibrinolysis, and endothelial function (85).

Of course, future studies might be based on panels other than those proposed in this study. Other promising biomarkers, such as glycogen phosphorylase isoenzyme BB (GPBB) (86), APOA1-UP (87), and platelet basic protein, have been described previously (88). These proteins have emerged as possible candidates showing high accuracy for distinguishing different conditions; however, more research is required to achieve the desired results of sensitivity and specificity during the process of validation.

### Limitations of Our Review

There are certain limitations to our study:

1. We only focused on studies conducted on IS caused by small-vessel disease due to the small number of studies reported in the literature regarding the subtype classification of stroke through biomarker combinations.
2. For the same reason, we evaluated only case–control studies, although this selection was intentional.
3. We did not carry out meta-analyses because of the high heterogeneity of the reported data and the wide variety of proteins used. Future studies must include statistical methods that ensure sufficient power to detect valid effects.
4. We could not identify a sufficient number of studies on biomarkers or panel biomarkers in stroke subtypes. Possible covariables interfering with the specificity of the biomarker [e.g., age, medications, lifestyle factors, and diseases; (16)] must also be incorporated into the statistical model.

However, this review highlights additional general methodological issues when studying stroke biomarker panels. These, of course, generate open questions that we have briefly discussed. We also comment on the design issues for testing the proposed panels.

### Considerations on the Statistical Methods in the Literature

Many studies do not provide complete information on the accuracy of diagnostic procedures to distinguish between two patient groups. Most articles report only sensitivity and specificity, which are threshold-dependent. We only included those providing the AUC in our review (89). See Supplementary Table 4 for the comparison. However, even this reporting level is insufficient because it is only useful to distinguish between the two groups. Critical clinical questions are therefore left unanswered when they require a distinction between several patient categories. To answer such questions involving three or more diagnostic groups, more sophisticated techniques are needed. Examples include the Youden index test proposed to generalize ROC curves by Obuchowski et al. (89) and Luo and Xiong (90).

### Unanswered Clinical Questions

- **Are biomarker panels useful for stratifying stroke risk levels?** For example, this question was examined by Laskowitz et al., who classified patients who applied logistic regression to a combination of biomarkers. These results showed that the odds ratio might be a good predictor of stroke risk. A similar evaluation was included in the evaluation protocol of the biomarker panels.
- **Are biomarker panels able to discriminate between small and large vessel strokes?** This question is crucial because these conditions require entirely different therapeutic or vascular surgical treatment approaches. Significantly, the accuracy of a biomarker panel might depend on this etiological difference. Unfortunately, few studies have addressed this issue quantitatively. In Table 4, we report the results of protein biomarkers with differential sensitivity to small-vessel disease and LVD. Note that our proposed panels include several biomarkers (as detailed in Supplementary Table 1), thus having the potential for this distinction.
- **Are biomarker panels able to discriminate CE and LVD stroke etiology?** Note that the accuracy of a biomarker panel might depend on the etiology of the IS. However, only a few studies have considered this issue (Table 4). The selected proteins for our proposed panels could have great potential because of their differential expression of serum levels in these stroke subtypes (Supplementary Table 3).
- **Are biomarker panels useful for the follow-up of mixed stroke cases?** Here, we refer to both hemorrhagic transformations of IS and secondary ischemic injury after ICH. Indeed, specific proteins have been explored in this context, as shown in Supplementary Table 2. These proteins have been included in our proposed panels, and studies to evaluate them will consider this aspect.

**Table 4:** Summary of the biomarkers for the determination of the subtypes of stroke.

**Biomarkers**	**Etiology**	**Sample/Methods**	**Cutoff/Time of blood drawing**	**Specificity**	**Sensitivity**	**References**
BNP	Cardioembolic **CE vs. other strokes subtypes**	200 patients (LVD = 18, CE = 82, SVD = 31, and other stroke = 69) Chemiluminescence enzyme immunoassay	140.0 pg/mL 24 h	80.5%, AUC = 0.87	80.5%, AUC = 0.87	(91)
BNP	Cardioembolic **CE vs. all stroke subtypes**	707 IS (LVD = 151, CE = 259, SVD = 128, and UE = 169) ELISA	76 pg/mL <24 h	69%	72%	(92)
BNP and DD	Cardioembolic **CE vs. all stroke subtypes**	707 IS (LVD = 151, CE = 259, SVD = 128, and UE = 169) ELISA	BNP>76 pg/mL DD> 0.96 μg/mL <24 h	91.3% AUC = 0.89	66.5% AUC = 0.89	(92)
D-Dimer	Cardioembolic **CE vs. all stroke subtypes**	707 IS (LVD = 151,CE = 259, SVD = 128, and UE = 169) ELISA	0.96 μg/mL <24 h	64%	56%	(92)
D-Dimer	Cardioembolic **CE vs. LVD + SVD**	126 IS (LVD = 34,CE = 34, SVD = 31, and UE = 27), and controls = 63 STA Liatest d-Dimer immunoassay (immunoturbidimetric technology)	2.00μg/mL >24 h	93.2%	59.3%	(93)
NT-proBNP	Cardioembolic **CE vs. all stroke subtypes**	114 IS (LVD = 27,CE = 34, SVD = 19, and UE = 34) Human RIAKit Phoenix Pharmaceuticals	200 pg/mL <6 h	82%	65%	(94)
Albumin/globulin ratio (G/A ratio)	Cardioembolic **CE vs. all stroke subtypes**	114 IS (LVD = 27,CE = 34, SVD = 19, and UE = 34) Immunoassay or colorimetric assay	0.7 <6 h	31%	91%	(94)
NT-proBNP and G/A ratio.	Cardioembolic **CE vs. all stroke subtypes**	114 IS (LVD = 27,CE = 34, SVD = 19, and UE = 34) Immunoassay or colorimetric assay	NT-proBNP >200 pg/mL G/A = 0.7 <6 h	AUC = 0.91 with Atrial Fibrillation (AF) AUC = 0.84 without AF		(94)
Pro-BNP	Cardioembolic **CE vs. all stroke subtypes**	262 IS (LVD = 44, CE = 100, SVD = 36, and UE = 82) Electrochemiluminescence immunoassay “ECLIA”	360 pg/mL <12 h	83% AUC = 0.921	87% AUC = 0.921	(95)
Pro-ANP	Cardioembolic **CE vs. all stroke subtypes**	262 IS (LVD = 44, CE = 100, SVD = 36, UE = 86) ELISA	2,266.6 fmol/mL <12 h	70% AUC = 0.735	62% AUC = 0.735	(95)
CK-MB	Cardioembolic **CE vs. all stroke subtypes**	262 IS (LVD = 44, CE = 100, SVD = 36, and UE = 86) Electrochemiluminescence immunoassay “ECLIA”	2.6 ng/mL <12 h	80% AUC = 0.731	62% AUC = 0.731	(95)
NT-proBNP	Cardioembolic **CE vs. no-CE**	Meta-analysis: six studies NT-proBNP prospective cohort	200–360 pg/mL <72 h	93% AUC = 0.87	55% AUC = 0.87	(96)
BNP	Cardioembolic **CE vs. no-CE**	Meta-analysis: ten studies BNP prospective cohort	64–155 pg/mL <24 h	85% AUC = 0.87	65% AUC = 0.87	(96)
Troponin	Embolic stroke of unknown source (ESUS) **ESUS vs. CE, non-CE**	1,120 IS [CE = 371, non-CE = 310, and embolic stroke of unknown source (ESUS) = 439] Sandwich immunoassay	ng/mL <24 h	95 %	12%	(97)
Troponin	Cardioembolic **CE vs. ESUS, non-CE**	1,120 IS [CE = 371, non-CE = 310, and embolic stroke of unknown source (ESUS) = 439] Sandwich immunoassay	ng/mL <24 h	95 %	17%	(97)
D-Dimer	SVD (lacunar) **SVD vs. CE + LVD**	126 patients (LVD = 34,CE = 34, SVD = 31, and UE = 27) STA Liatest d-Dimer immunoassay (immunoturbidimetric technology)	0.54 μg/mL >24 h	96.2%	61.3%	(93)
Homocysteine (Hcy)	Lacunar (SVD) **SVD vs. controls**	197 acute lacunar infarction patients and 192 controls –	15.5 μmol/L <24 h	100% AUC = 0.881	65% AUC = 0.881	(98)
Fibrinogen	Lacunar (SVD) **SVD vs. controls**	197 acute lacunar infarction patients and 192 controls –	228.55 μg/dL <24 h	58.3% AUC = 0.688	83.2% AUC = 0.688	(98)
Hcy/fibrinogen	Lacunar (SVD) **SVD vs. controls**	197 acute lacunar infarction patients and 192 controls –	15.5 μmol/L 228.55 μg/dL <24 h	58.3% AUC = 0.766	94.9% AUC = 0.766	(98)
GFAP/d-dimer preprint	LVD **LVD vs. other strokes**	128 patients (LVD = 23, non-LVD = 42, HS = 16, stroke mimic = 31, and TIA = 16) ELISA	d-dimer +GFAP = 0.33	92% AUC = 0.81	57% AUC = 0.81	(99)

*Bold values indicate groups compared in the studies analyzed by Receiver Operating Curve analysis (ROC)*.

We hope that this review will stimulate additional proposals of other biomarker panels that might contribute to the long-term objective of stroke precision medicine. We emphasize again that we have concentrated on biomarkers obtainable from plasma at a low cost with scalable technologies in any economic setting. If not these panels of biomarkers for stroke, then similar ones might be the key to tackling the global burden of disease due to stroke.

## Conclusions

More research is needed to validate, identify, and introduce into medical practice useful biomarkers for stroke recurrence or diagnosis in a scalable manner. The most promising approach is to combine a panel of different blood-based proteins to provide acceptable diagnostic precision for health interventions. After a systematic review, we suggest three novel biomarker panels based on the results in the literature with an interpretation based on stroke pathophysiology.

## Data Availability Statement

The original contributions presented in the study are included in the article/Supplementary Material, further inquiries can be directed to the corresponding author/s.

## Author Contributions

SB, JL, and PV-S: study concept and design and drafted the manuscript. AH-S, DG, and SB: acquisition, analysis, or interpretation of the data. PV-S and GG administered the project. PV-S and MB-V supervised the study. All authors critically revised the manuscript. All authors had full access to all the data in the study. They take responsibility for the integrity and accuracy of the analysis and results.

## Conflict of Interest

The authors declare that the research was conducted in the absence of any commercial or financial relationships that could be construed as a potential conflict of interest.

## Abbreviations

AIS: acute ischemic stroke
IS: ischemic stroke
ICH: intracerebral hemorrhage
TIA: transient ischemic attack
SAH: subarachnoid hemorrhage
VCAM: vascular cell adhesion molecule
vWF: von Willebrand factor
BNGF: B-type neurotrophic growth factor
CRP: C-reactive protein
sRAGE: soluble receptor for advanced glycation end products
MMP-9: matrix metalloproteinase 9
BNP: brain natriuretic peptide
TIMP-4: metalloproteinase inhibitor-4
UCH-L1: ubiquitin C-terminal hydrolase 1
CE: cardioembolic stroke subtype
LVD: large-vessel disease stroke subtype
SVD: small-vessel disease
UDE: undetermined etiology
ELISAs: enzyme-linked immunosorbent assay.
