# Supplementary material for: Scalable Bio Marker Combinations for Early Stroke Diagnosis: A Systematic Review
Source: Front Neurol. 2021 May 28;12:638693. doi: 10.3389/fneur.2021.638693 (PMC8193128; doi:10.3389/fneur.2021.638693)
Supplement: Supplementary file 1 [file Data_Sheet_1.PDF]

SUPLEMENTARY INFORMATION

Table S-1 Biomarkers derived from the biomarker panel proposal and their relation to Small Vessel Disease and Large Vessel Disease Subtypes

Proteins	Small Vessel Disease	Large Vessel Disease	Ref.
NR2			
GFAP		+	[1]
MMP-9	+	+	[2], [3]–[5], [6]
vWF	+	+	[7]–[9], [10], [11]
S100β			
IMA index			
Albumin	+	+	[12], [13]
AT-III	+		[14]
Fibrinogen	+	+	[15]–[17]

Table S-2 Biomarkers derived from the biomarker panel proposal and their relation to Hemorrhagic Transformation and Secondary Ischemic Injury

Proteins	Hemorrhagic Transformation	Secondary Ischemic Injury	References
NR2		+	[18]
GFAP		+	[19]
MMP-9	+	+	[20], [21], [22]
vWF	+	+	[23], [35]
S100β	+	+	[24], [25]
IMA index			
Albumin	+		[26], [38]
AT-III		+	[27], [43]
Fibrinogen	+	+	[18][28], [29], [30], [31]

Table S-3 Biomarkers derived from the biomarker panel proposal related to Large Vessel Disease and Cardio-embolic Subtype.

Proteins	Large Vessel Disease	Cardio-embolic	Ref.
NR2			
GFAP	+		[1]
MMP-9	+	+	[2], [3]–[5], [6]
vWF	+	+	[7]–[9], [10], [11]
S100β			
IMA index		+	[32]
Albumin	+	+	[12], [13]
AT-III		+	[33],[14]
Fibrinogen	+	+	[15]–[17]

Table S-4 Groups evaluated by ROC analysis derived from articles included in the systematic review

Groups evaluated by ROC Curves	Number
IS vs. mimics	3
ICH vs. mimics	1
AIS vs. others	4
IS + ICH vs. mimics	1
IS vs. ICH	3
ICH vs. others	1
ICH vs. Control	1

REFERENCES FOR SUPPLEMENTARY TABLES:

[1] A. E. Gaude *et al.*, “Combination of blood biomarkers and stroke scales improves identification of large vessel occlusions,” *Prepr. medRxiv*, 2021.

[2] K. Buraczynska, J. Kurzepa, A. Ksiazek, M. Buraczynska, and K. Rejdak, “Matrix Metalloproteinase-9 (MMP-9) Gene Polymorphism in Stroke Patients,” *NeuroMolecular Med.*, vol. 17, no. 4, pp. 385–390, 2015, doi: 10.1007/s12017-015-8367-5.

[3] I. Vukasovic, A. Tesija-Kuna, E. Topic, V. Supanc, V. Demarin, and M. Petrovcic, “Matrix metalloproteinases and their inhibitors in different acute stroke subtypes,” *Clin. Chem. Lab. Med.*, vol. 44, no. 4, pp. 428–434, 2006, doi: 10.1515/CCLM.2006.079.

[4] J. Montaner *et al.*, “Etiologic diagnosis of ischemic stroke subtypes with plasma biomarkers,” *Stroke*, vol. 39, no. 8, pp. 2280–2287, 2008, doi: 10.1161/STROKEAHA.107.505354.

[5] M. F. Lehmann *et al.*, “Inflammatory and metabolic markers and short-time outcome in patients with acute ischemic stroke in relation to TOAST subtypes.,” *Metab. Brain Dis.*, vol. 30, no. 6, pp. 1417–1428, Dec. 2015, doi: 10.1007/s11011-015-9731-8.

[6] I. M. Loftus *et al.*, “Increased matrix metalloproteinase-9 activity in unstable carotid plaques. A potential role in acute plaque disruption.,” *Stroke*, vol. 31, no. 1, pp. 40–47, Jan. 2000, doi: 10.1161/01.str.31.1.40.

[7] P. Cherian *et al.*, “Endothelial and platelet activation in acute ischemic stroke and its etiological subtypes,” *Stroke*, vol. 34, no. 9, pp. 2132–2137, 2003, doi: 10.1161/01.STR.0000086466.32421.F4.

[8] M. A. H. Sonneveld *et al.*, “Relationship of Von Willebrand Factor with carotid artery and aortic arch calcification in ischemic stroke patients,” *Atherosclerosis*, vol. 230, no. 2, pp. 210–215, 2013, doi: 10.1016/j.atherosclerosis.2013.07.046.

[9] W. O. Tobin *et al.*, “Profile of von Willebrand factor antigen and von Willebrand factor propeptide in an overall TIA and ischaemic stroke population and amongst subtypes.,” *J. Neurol. Sci.*, vol. 375, pp. 404–410, Apr. 2017, doi: 10.1016/j.jns.2017.02.045.

[10] E. Hanson, K. Jood, S. Karlsson, S. Nilsson, C. Blomstrand, and C. Jern, “Plasma levels of von Willebrand factor in the etiologic subtypes of ischemic stroke,” *J. Thromb. Haemost.*, vol. 9, no. 2, pp. 275–281, 2011, doi: 10.1111/j.1538-7836.2010.04134.x.

[11] M. Menih, M. Križmarić, and T. Hojs Fabjan, “Clinical role of von Willebrand factor in acute ischemic stroke,” *Wien. Klin. Wochenschr.*, vol. 129, no. 13–14, pp. 491–496, 2017, doi: 10.1007/s00508-017-1200-4.

[12] H. Naito *et al.*, “Prognostic role of the controlling nutritional status score in acute ischemic stroke among stroke subtypes,” *J. Neurol. Sci.*, vol. 416, no. June, p. 116984, 2020, doi: 10.1016/j.jns.2020.116984.

[13] W. H. Xu, C. Dong, T. Rundek, M. S. V. Elkind, and R. L. Sacco, “Serum albumin levels are associated with cardioembolic and cryptogenic ischemic strokes: Northern Manhattan study,” *Stroke*, vol. 45, no. 4, pp. 973–978, 2014, doi: 10.1161/STROKEAHA.113.003835.

[14] K. Hirano *et al.*, “Study of hemostatic biomarkers in acute ischemic stroke by clinical subtype,” *J. Stroke Cerebrovasc. Dis.*, vol. 21, no. 5, pp. 404–410, 2012, doi: 10.1016/j.jstrokecerebrovasdis.2011.08.013.

[15] F. J. Alvarez-Perez, M. Castelo-Branco, and J. Alvarez-Sabin, “Usefulness of measurement of fibrinogen, D-dimer, D-dimer/fibrinogen ratio, C reactive protein and erythrocyte sedimentation rate to assess the pathophysiology and mechanism of ischaemic stroke.,” *J. Neurol. Neurosurg. Psychiatry*, vol. 82, no. 9, pp. 986–992, Sep. 2011, doi: 10.1136/jnnp.2010.230870.

[16] W. Turaj *et al.*, “Increased plasma fibrinogen predicts one-year mortality in patients with acute ischemic stroke,” *J. Neurol. Sci.*, vol. 246, no. 1–2, pp. 13–19, 2006, doi: 10.1016/j.jns.2006.01.020.

[17] H. Fan *et al.*, “Assessment of Homocysteine as a Diagnostic and Early Prognostic Biomarker for Patients with Acute Lacunar Infarction.,” *Eur. Neurol.*, vol. 79, no. 1–2, pp. 54–62, 2018, doi: 10.1159/000484893.

[18] A. S. Brunswick, B. Y. Hwang, G. Appelboom, R. Y. Hwang, M. A. Piazza, and E. S. Connolly, “Serum biomarkers of spontaneous intracerebral hemorrhage induced secondary brain injury,” *J. Neurol. Sci.*, vol. 321, no. 1–2, pp. 1–10, 2012, doi: 10.1016/j.jns.2012.06.008.

[19] A. Petzold *et al.*, “Early identification of secondary brain damage in subarachnoid hemorrhage: A role for glial fibrillary acidic protein,” *J. Neurotrauma*, vol. 23, no. 7, pp. 1179–1184, 2006, doi: 10.1089/neu.2006.23.1179.

[20] J. Montaner *et al.*, “Matrix metalloproteinase-9 pretreatment level predicts intracranial hemorrhagic complications after thrombolysis in human stroke,” *Circulation*, vol. 107, no. 4, pp. 598–603, 2003, doi: 10.1161/01.CIR.0000046451.38849.90.

[21] M. Castellanos *et al.*, “Plasma metalloproteinase-9 concentration predicts hemorrhagic transformation in acute ischemic stroke.,” *Stroke*, vol. 34, no. 1, pp. 40–46, Jan. 2003.

[22] M. J. McGirt *et al.*, “Serum von Willebrand factor, matrix metalloproteinase-9, and vascular endothelial growth factor levels predict the onset of cerebral vasospasm after aneurysmal subarachnoid hemorrhage,” *Neurosurgery*, vol. 51, no. 5, pp. 1128–1135, 2002, doi: 10.1097/00006123-200211000-00005.

[23] N. K. Tóth *et al.*, “Elevated factor VIII and von Willebrand factor levels predict unfavorable outcome in stroke patients treated with intravenous thrombolysis,” *Front. Neurol.*, vol. 8, no. JAN, pp. 1–10, 2018, doi: 10.3389/fneur.2017.00721.

[24] M. Herrmann, P. Vos, M. T. Wunderlich, C. H. M. M. de Bruijn, and K. J. B. Lamers, “Release of Glial Tissue–Specific Proteins After Acute Stroke,” *Stroke*, vol. 31, no. 11, pp. 2670–2677, 2000, doi: 10.1161/01.str.31.11.2670.

[25] C. S. Jung, B. Lange, M. Zimmermann, and V. Seifert, “CSF and Serum Biomarkers Focusing on Cerebral Vasospasm and Ischemia after Subarachnoid Hemorrhage.,” *Stroke Res. Treat.*, vol. 2013, p. 560305, 2013, doi: 10.1155/2013/560305.

[26] R. Che *et al.*, “Low Serum Albumin level as a Predictor of Hemorrhage Transformation after Intravenous Thrombolysis in Ischemic Stroke Patients.,” *Sci. Rep.*, vol. 7, no. 1, p. 7776, Aug. 2017,

doi: 10.1038/s41598-017-06802-y.

[27] K. R. Lee, N. Kawai, S. Kim, O. Sagher, and J. T. Hoff, “Mechanisms of edema formation after intracerebral hemorrhage: Effects of thrombin on cerebral blood flow, blood-brain barrier permeability, and cell survival in a rat model,” *J. Neurosurg.*, vol. 86, no. 2, pp. 272–278, 1997, doi: 10.3171/jns.1997.86.2.0272.

[28] R. Wang, J. Zeng, F. Wang, X. Zhuang, X. Chen, and J. Miao, “Risk factors of hemorrhagic transformation after intravenous thrombolysis with rt-PA in acute cerebral infarction,” *Qjm*, vol. 112, no. 5, pp. 323–326, 2019, doi: 10.1093/qjmed/hcy292.

[29] L. Vandelli *et al.*, “Fibrinogen decrease after intravenous thrombolysis in ischemic stroke patients is a risk factor for intracerebral hemorrhage,” *J. Stroke Cerebrovasc. Dis.*, vol. 24, no. 2, pp. 394–400, 2015, doi: 10.1016/j.jstrokecerebrovasdis.2014.09.005.

[30] M. E. Barra *et al.*, “Fibrinogen Concentrate for the Treatment of Thrombolysis-Associated Hemorrhage in Adult Ischemic Stroke Patients,” *Clin. Appl. Thromb.*, vol. 26, 2020, doi: 10.1177/1076029620951867.

[31] Y. Ruan *et al.*, “High fibrinogen-to-albumin ratio is associated with hemorrhagic transformation in acute ischemic stroke patients,” *Brain Behav.*, no. September, pp. 1–9, 2020, doi: 10.1002/brb3.1855.

[32] M. K. Sinha, D. C. Gaze, J. R. Tippins, P. O. Collinson, and J. C. Kaski, “Ischemia modified albumin is a sensitive marker of myocardial ischemia after percutaneous coronary intervention,” *Circulation*, vol. 107, no. 19, pp. 2403–2405, 2003, doi: 10.1161/01.CIR.0000072764.18315.6B.

[33] K. Takano, T. Yamaguchi, H. Kato, and T. Omae, “Activation of coagulation in acute cardioembolic stroke,” *Stroke*, vol. 22, no. 1, pp. 12–16, 1991, doi: 10.1161/01.STR.22.1.12.
